# Network centrality for the identification of biomarkers in respondent-driven sampling datasets

**DOI:** 10.1371/journal.pone.0256601

**Published:** 2021-08-24

**Authors:** Jacob Grubb, Derek Lopez, Bhuvaneshwar Mohan, John Matta

**Affiliations:** Computer Science Department, Southern Illinois University Edwardsville, Edwardsville, IL, United States of America; Unviersity of Burgundy, FRANCE

## Abstract

Networks science techniques are frequently used to provide meaningful insights into the populations underlying medical and social data. This paper examines SATHCAP, a dataset related to HIV and drug use in three US cities. In particular, we use network measures such as betweenness centrality, closeness centrality, and eigenvector centrality to find central, important nodes in a network derived from SATHCAP data. We evaluate the attributes of these important nodes and create an exceptionality score based on the number of nodes that share a particular attribute. This score, along with the underlying network itself, is used to reveal insight into the attributes of groups that can be effectively targeted to slow the spread of disease. Our research confirms a known connection between homelessness and HIV, as well as drug abuse and HIV, and shows support for the theory that individuals without easy access to transportation are more likely to be central to the spread of HIV in urban, high risk populations.

## Introduction

In this paper we utilize several types of network centrality measures, including betweenness, closeness, and eigenvalue, to find nodes central to the structure of the SATHCAP Referral Network, a network based on concurrent drug use and sexual activity. Epidemiological theory suggests that interventions targeting these central nodes are more effective in stopping the spread of a disease through a network than interventions involving other nodes [1, 2]. By finding and examining these central nodes, we hope to find a set of shared attributes, or biomarkers. Knowledge of these biomarkers can be used to design targeted intervention campaigns to make diseases such as HIV less likely to spread through high risk environments.

The importance of learning about HIV transmission via tools such as network science is great. HIV began as an epidemic in 1981 and continues to impact many segments of society, particularly populations considered *hidden* or *hard-to-reach*, such as minorities and men who have sex with men (MSM). The disease remains stigmatized, such that 1 in 7 people who have HIV do not know it [3], and half of minority MSM will become HIV-infected in their lifetime [4]. Minority women suffer worse health outcomes with HIV than other women [5].

Surveys conducted using respondent-driven sampling (RDS) attempt to discover information specifically about a hidden population through the use of peer recruitment to find participants [6]. The SATHCAP RDS [7] focuses on low-income, minority MSM and injected drug users. A large amount of data was collected from each participant, making this study a particularly rich source of information about a hidden population that is likely to be affected by HIV. The SATHCAP study was conducted between 2006 and 2008 in the US cities of Chicago, Los Angeles, and Raleigh, NC. The 4,688 participants were asked almost 1500 questions concerning their sexual and drug-related habits, as well as demographic and other information. The nature of the RDS sampling in this study is that new recruits had participated in potential HIV-spreading behavior with their recruiter. The derived network is therefore representative of one through which HIV and other diseases could have spread. Our methodology finds the three largest connected components within each city and looks for shared attributes between the central-most nodes within these components.

If properties of actors susceptible to HIV were analyzed through a statistically random survey of the general population, it is likely that few members of the hidden populations of interest would be found, because of overall low prevalences. Use of an RDS survey has two benefits. First, sampling only the hidden population allows an analysis within that population, giving more detailed results when determining attributes of high risk individuals. Second, an RDS survey preserves the connections between individuals, which is another source of information that is lost in a large independent survey.

In an RDS survey the initial samples (called *seeds*) are not random, but if there are enough recruitment waves the surveyed population becomes statistically independent of the seeds [6]. An RDS survey relies on *homophily* in recruitment, which is the property that people in the studied hidden populations know each other (while remaining mostly unknown to researchers). Because of homophily, the construction of the SATHCAP network is not necessarily random [8], but instead has a higher probability of recruitment of the target population. The RDS network obtained is a series of trees, which is a by-product of the recruitment process. While the underlying network may have loops, studies have shown that sexual networks have few loops [9], including the HIV transmission network of a large US city [10]. Because the network underlying SATHCAP is likely scale free [11] (as are many sexual networks, for example [12]), high degree nodes (or hubs) are important. The probability that the RDS sample contains hubs is great—much greater than the probability that they would be included in a general-population random sample.

Finding and helping vulnerable hidden populations has previously shown great benefits. Due to extensive preventative efforts, HIV diagnosis rates from 2010 to 2015 decreased in the categories of MSM and persons who inject drugs. However, diagnosis rates still increased in the more specific (and hidden or hard-to-reach) categories of black individuals and MSM aged 25-34 [13]. Recent studies have shown that viral suppression (achieved through highly active antiretroviral therapy, or HAART) prevents sexual transmission of HIV [14]. Once at-risk groups are identified, such as through network science techniques, preventative interventions can be attempted such as HAART for infected individuals, PrEP for uninfected individuals [15], and educational interventions for all.

This paper is an extension of [11]. The previous paper used only betweenness centrality as a metric for importance. This paper improves upon the previous paper by also examining closeness centrality and eigenvector centrality. Each method of centrality discovers a different set of central nodes, each of which has their own set of notable attributes. We further improve upon the previous work by distributing the set of central attributes between the three largest components, taking the top five central nodes from each component to yield a total of 15 nodes, rather than taking the top 10 most central nodes from each city. Because of the homophily involved in the data collection, nearby nodes within the same component are more likely to share similar attributes. Choosing nodes from different components prevents the largest components from overwhelming the other large components and gives a more diverse sample of central nodes across the city networks.

The data have been obtained through the National Addiction and HIV Data Archive Program (NAHDAP), accessible online https://www.icpsr.umich.edu/icpsrweb/NAHDAP/index.jsp. This research was conducted under the approval of the Southern Illinois University Edwardsville IRB.

## Related work

The SATHCAP dataset was the basis for several papers published in a special issue of the Journal of Urban Health in 2009 [7]. One paper from that special issue that focuses on network interpretation is [16]. In that paper Youm et al. identify sets of “bridging individuals” between communities in Chicago. That paper also identifies hidden communities that have an impact on the spread of the HIV despite low disease incidence rates. Because of their low overall incidence rates, these communities would not have been discovered through a general population survey. However, targeted interventions within these specific communities would materially reduce the transmission rate through the entire city of Chicago. In other work, Ober et al. [17] use the SATHCAP dataset to identify factors associated with stimulant drug use during sexual activity between older, low income males.

Network analysis of spreading has been conducted in many contexts, such as in terms of social contacts [18] and rodent infestations [19], among others. Because contacts are generally known and can be traced, network analysis is particularly suited to sexually transmitted diseases, such as gonorrhoea [20] and chlamydia [21] in addition to HIV [22]. Liljeros et al. [23] provide an introduction into the use of network theory to describe sexual interactions and the application of this theory to the spread of venereal diseases. An early example of the use of network theory to represent the spread of HIV can be found in [24], which provides a series of mathematical formulas to predict the spread of disease in heterogeneous sexual networks.

Graph-based aspects of respondent driven sampling, and the fact that “data collected in ordinary RDS studies contain information about the structure of the respondents’ social network” are discussed in [25]. The three network centrality measures considered in this paper are well-known and have previously been used many times in the context of analyzing disease spread. Betweenness centrality is often used to find topologically important nodes relating to network resilience and spreading [26], including the spread of diseases like HIV [27]. Closeness centrality has been found to be influential in models of disease spreading in pig populations [28]. In [29] it is found that SARS-CoV-2 spreads to Brazilian cities more quickly based on the closeness centrality of the city’s airport. Eigenvector centrality is used in many instances to study the spread of disease in epidemics [30, 31], and in [32] it is shown that “eigenvector centrality approximately quantifies the risk of a node to become infected” with COVID-19. In [33] it is shown that the eigenvector centrality values of the seed nodes in an Italian cattle network are strongly correlated with the extent of the spread of an epidemic through the network. In a bioinformatics example of its use, eigenvector centrality has connections to brain network alterations in Alzheimer’s disease [34].

Respondent driven sampling (RDS) was first presented as part of an AIDS prevention initiative, as a method for interviewing subjects independent of the original sample subject [6]. By utilizing chains of peer recruitment combined with Markov modeling, RDS is able to access remote populations while theoretically reducing sampling bias to a reliably low level [35].

## Methodology

### Respondent driven sampling

RDS is a data collection technique in which samples are generated from a random walk along nodes in the underlying network. Sampling probability is proportional to the node’s degree [36]. There are several assumptions necessary to ensure the independence of samples in the RDS process. The members of populations sampled must be able to identify each other, and preferably will have acquaintanceships that form a connected network. The size of data collected must be small relative to the overall size of the population such that the pool of recruits is not quickly exhausted. Participants must be able to choose new recruits randomly, and participants must be able to accurately report the number of acquaintanceships they have. The weakness of RDS lies in the degree to which these assumptions may be false [8]. The strength of RDS is in obtaining information about hidden populations that may be impossible to obtain through standard random surveys.

It is shown in [11] that the underlying network of SATHCAP is scale free. This implies that the network has hubs [37], and the distance from a random node to a hub in a scale free network is short (in theory, of the order of the log of the size of the network [38]). RDS (and snowball-style sampling methods in general) tends to choose hubs [39, 40]. This fact is used in the analysis of RDS data. For example, Successive Sampling Population Size Estimation (SS-PSE) is a technique developed by Gile and Handcock [36] for estimating the size of the underlying network based on an RDS sample. This technique assumes that high degree nodes are sampled early in the process, and if high degree nodes remain after several waves of recruitment, the size of the hidden population must be correspondingly larger. Also, because of its propensity to select hubs, RDS has been suggested as a way to find high degree nodes for immunization [41].

### Data parsing and cleaning

The original SATHCAP dataset contained a total of 4688 participants who were asked 1493 questions. The answers given are referred to here as *features* or *attributes* of the participants. Peer recruitment into the study happened via coupons which were given to seed participants to distribute to possible study recruits. The coupons were color-coded, based on the relationship between the recruiter and recruitee, with different colors representing sexual connections and drug-using connections. Information from the coupon numbers, city codes, and colors were used to create unique identifiers for each participant. This enabled each participant to be traced to a position in the recruitment network.

Due to the length of the survey and the nature of the questions, respondents were given the option to decline to answer questions. This meant that there was a great deal of missing data. Over 40% of features (n = 1352/1488) are missing more than 40% of observations. Features missing more than a given threshold percentage of observations were removed, where the optimal threshold has a high number of features with a low percentage of missing observations. Features having at least 94% of observations were retained, except features consisting of metadata. This left a total of 80 features.

One-hot encoding was used to convert attributes without an ordinal relationship into separate binary attributes. For example, a question about income with possible answers of *high*, *medium*, and *low* is converted into three questions with yes/no answers: *Is income high?, Is income medium?, Is income low?*. Thus the answers to the questions become features of the participants, such as *low income*. The end result after one-hot encoding of multi-valued attributes was a set of 141 features.

### City network creation

The city networks were created using the participant identifier numbers. Each participant is a node in the network, and links were created between each node and the nodes it recruited. The result is a forest of tree graphs. Many of the seed nodes did not recruit additional participants. In fact, 255 out of 412 connected components in the final network are of size 1 or 2, indicating a situation in which a seed node recruited no one or only 1 participant. Successful chains of recruitment have between 30 and 949 nodes. For our analysis, we look at the three largest components within Los Angeles, Chicago, and Raleigh-Durham, for a total of 9 distinct graphs across three cities. Due to the structure of recruitment, there are no cycles in the network (a participant could not recruit someone who had already been recruited), and the maximum degree of a node is 7 (a participant could recruit a maximum of 6 others).

### Calculation of centrality

**Fig 1:**
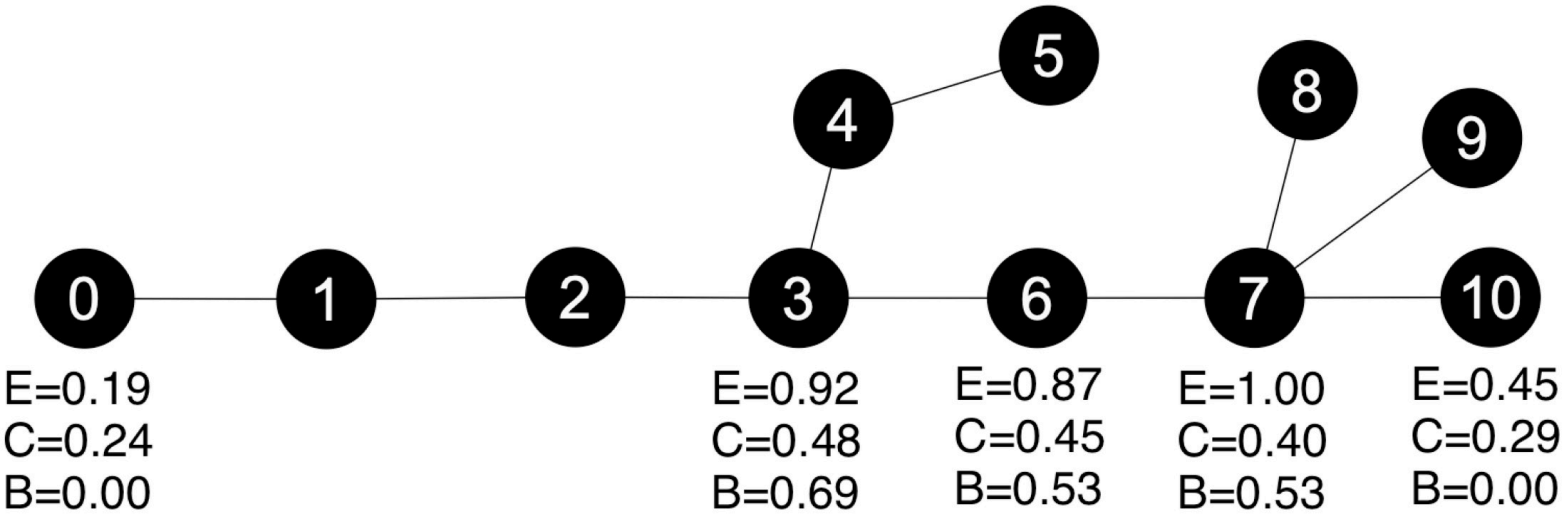
Values for different centrality measures are shown for nodes in a tree. *E* represents eigenvector centrality, *C* is closeness centrality, and *B* is betweenness centrality.

As an improvement upon [11], we include two new forms of centrality in addition to the previous betweenness centrality. This work includes the use of closeness centrality and eigenvector centrality as metrics of importance. Each of these centrality measures is concerned with different network properties. Calculated example values for a simple tree network are shown in Fig 1.

The eigenvector centrality of a node is based on the node’s degree, and is a numerical representation of the “popularity” of a node, where nodes that are connected with other “popular” or high degree nodes are given higher centrality scores. As such, it is a useful measure for determining highly influential (and influenced) nodes in social networks [42, 43]. This measure is calculated by finding the principal eigenvector of the adjacency matrix of the graph. By finding “popular” nodes within SATHCAP, we highlight sets of nodes with a high probability of infection based on their proximity to infected nodes. Eigenvector centrality does this without relying exclusively on degree centrality, which is problematic on a graph whose degree is artificially limited, such as SATHCAP. In the Fig 1 example, nodes 7 and 3 have the highest eigenvector centrality, and are also the highest degree nodes. Despite having a low degree, node 6 has a high eigenvector centrality because of its proximity to nodes 7 and 3. Nodes 8, 9 and 10 have a medium eigenvector centrality, despite having a low degree of 1, based only their proximity to high-degree node 7. Compare this to node 0, also with degree 1, which has a low eigenvector centrality. It can be seen that eigenvector centrality is a meaningful measure, even in a small tree example.

The closeness centrality [44] of a node is defined as the inverse of the sum of distance of the shortest paths from that node to every other node in the connected component. Mathematically, normalized closeness centrality is expressed as

$$\begin{array}{c} c\left(v\right)=\frac{N}{\sum_{y}d\left(y,v\right)} \end{array}$$ (1)

where N is the number of nodes, and *d*(*y*, *v*) represents the distance from node *y* to node *v*. Closeness centrality is useful for identifying nodes that are near to other nodes. Within SATHCAP, these nodes would represent a hazard if infected, as the short distance to every other node would potentially represent an easier path for transmission. In Fig 1 nodes 3 and 6 have the highest closeness centrality. Node 7 has a comparatively lower closeness centrality, despite its high degree. Node 0 is furthest from other nodes and therefore has the lowest closeness centrality.

The betweenness centrality [45, 46] of a node is based on the shortest paths property of a graph, and is defined as the proportion of paths that traverse that node when calculating the shortest paths from all nodes to all other nodes. A mathematical formula describing this measure can be seen in Eq 2, where *σ*_*st*_ represents the total number of shortest paths from node *s* to node *t* and *σ*_*st*_(*v*) represents the number of those paths that contain node *v*.

$$\begin{array}{c} b\left(v\right)=\sum_{s\neq v\neq t}\frac{\sigma_{st}\left(v\right)}{\sigma_{st}} \end{array}$$ (2)

Betweenness centrality is a valuable metric within the SATHCAP respondent driven sampling network, as it highlights the nodes that lie on the shortest and most direct paths for the transmission of disease. Node 3 has the highest betweenness centrality in Fig 1. As an example of the differences of the three centrality measures, note that while nodes 8, 9, and 10 have relatively high eigenvector centrality, they do not lie on any transmission paths, and therefore have a betweenness centrality of zero.

### Identification of central nodes

After calculating the betweenness, closeness, and eigenvector centrality scores for each node within the SATHCAP referral network, we need to identify the nodes that are most central.

In [11], we used the trivial solution of taking the ten nodes with the highest score in each city. While this is logical, difficulties arise when plotting these nodes on the underlying graph. Due to large discrepancies in the size of components, many to all of the central nodes would appear in a single component, frequently connected together in a chain. This methodology resulted in discarding other large, important components that were overshadowed by the size of the largest component.

To combat this overshadowing, we instead take our central nodes from a variety of components. We find the five nodes with the highest centrality scores from each of the three largest components in each city. This yields a total of 15 central nodes from each city, better distributed throughout each of the large components.

### Calculation of attribute exceptionality

Once the set of central nodes, *c*, within each city has been identified, we examine the attributes, *i*, of the respondent associated with each central node. We compare the value of each attribute to the average value of that attribute for the city it belongs to. The ‘city average’ for each variable is taken to be the average value of all respondents from that city within the SATHCAP dataset. We note if an attribute appears more than two standard deviations away than the city average, indicating, based on a 95% confidence interval, that the value is an outlier from the average response. If an attribute appears deviant in a large number (at least 4 of 15) of central nodes, we consider that attribute to be “exceptional,” and give it an exceptionality score equal to the number of central nodes that possessed that deviant attribute, *c*_*i*_, divided by the total number of central nodes, such that $exceptionality=\frac{|c_{i}|}{\left|c\right|}$. We repeat this calculation for each city and centrality type to find a set of attributes for each city-centrality combination.

## Results

**Fig 2:**
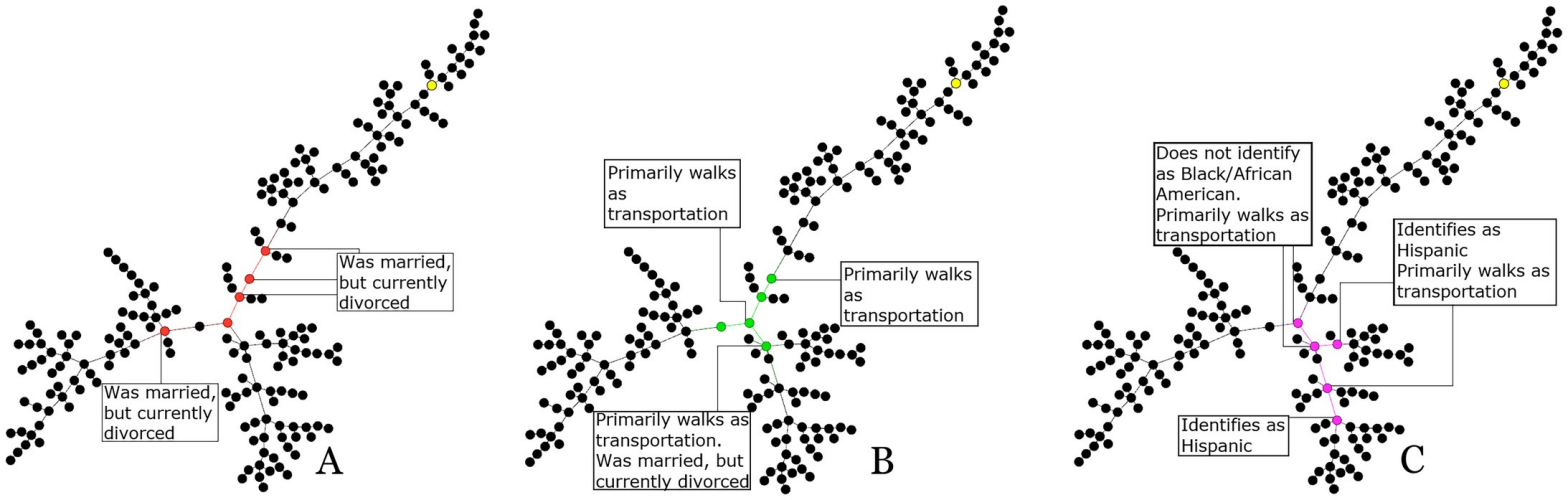
One component from the Chicago referral network, with nodes highlighted by centrality. **A**. Betweenness. **B**. Closeness. **C**. Eigenvector.

**Fig 3:**
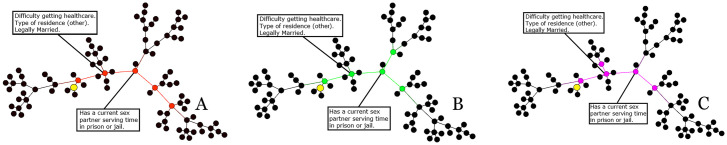
One component from the Los Angeles referral network, with nodes highlighted by centrality. **A**. Betweenness. **B**. Closeness. **C**. Eigenvector.

**Fig 4:**
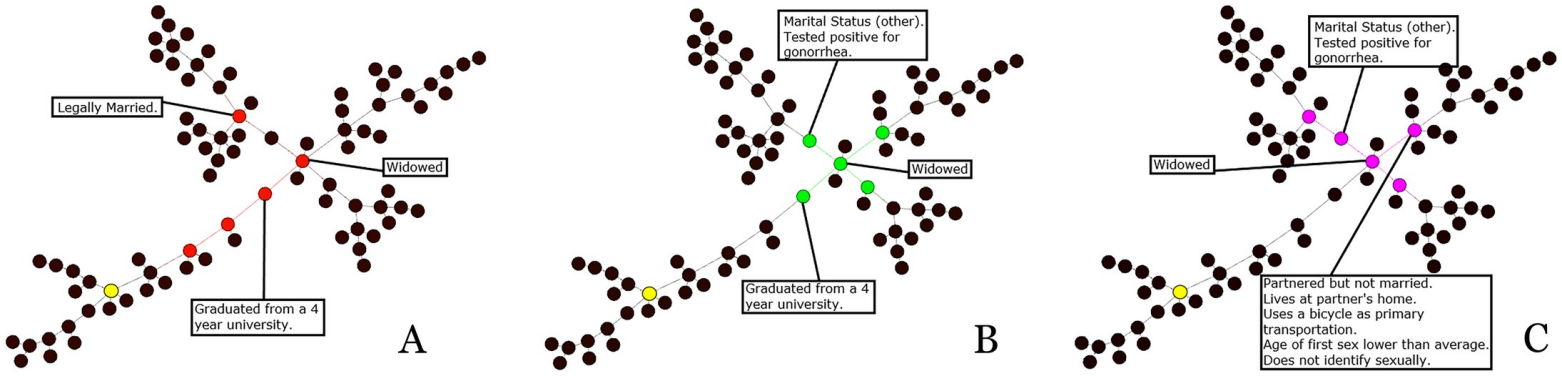
One component from the Raleigh-Durham referral network, with nodes highlighted by centrality. **A**. Betweenness. **B**. Closeness. **C**. Eigenvector.

Figs 2–4 highlight the nodes identified by each centrality method within a single component of each city. It is apparent that there are differences in the set of nodes identified by each centrality method. High betweenness nodes are highlighted in red, nodes with high closeness centrality are green, and nodes with high eigenvector centrality are purple. The seed node for each component is yellow. Each subfigure is labeled by its corresponding centrality method. By looking at the shared sets of attributes within these nodes and counting the number of times those attributes fall more than two standard deviations from the city average, we find a set of shared, exceptional attributes that best describe the central nodes.

The three centrality methods were run on the three largest connected components in the Chicago network. Results for one of the Chicago components are shown in Fig 2. In this example, each centrality method found a different set of nodes, although all sets were found close to the connection point of the three distinct branches. Interestingly, the seed node is towards the end of a branch and is not at all central. Closeness centrality, as seen in Fig 2B, does the best job at centering the nodes around this connection point, while betweenness and eigenvector select different nodes on branches. Across each graph, we can see nodes highlighted with relevant exceptional attributes. Some attributes, such as the respondent’s marital status or primary mode of transportation appeared across multiple types of centrality, while other attributes such as the subject’s ethnic background were only found to be exceptional in a single centrality method, as seen in the eigenvector nodes highlighted in Fig 2C.

Complete results for the three largest components within the Chicago referral network are shown in Table 1. Attributes such as ‘mstat’, which refers to marital status, and ‘tmode’ which refers to transportation mode, are shared between multiple centrality methods, indicating a higher concentration of individuals with those attributes within the most central nodes of the network. While other attributes, such as ‘hisp’ and ‘raceb’, which concern identification as Hispanic or African American, are found to be exceptional within the eigenvector nodes, this observation is not shared between other forms of centrality, indicating this attribute is less likely to be important to the structure of the overall network.

**Table 1:** Exceptional attributes of Chicago.

Centrality Type	Attribute Code	Attribute Plaintext	Exceptionality
betweenness	mstat-4	Married but currently separated	0.2667
closeness	mstat-5	Currently Divorced	0.3333
	tmode-5	Walks as a primary form of transportation	0.2667
eigenvector	tmode-5	Walks as a primary form of transportation	0.4667
	raceb	Less likely to identify as Black or African American	0.3333
	hisp	More likely to identify as Spanish, Hispanic, or Latino	0.2667

One of the Los Angeles Network components is shown in Fig 3. Here we see a similar structure to Chicago, with several branches extending from a central set of nodes. Unlike Chicago however, we notice significantly more overlap in the sets of nodes produced by each centrality method. This overlap results in a set of attributes identified by each centrality method as exceptional, albeit to varying degrees. As shown in Table 2, ‘reside-5’, indicating living situation, and ‘used-i’, indicating drug use, have consistent exceptionality scores ranging between 0.267 and 0.333 across each method.

**Table 2:** Exceptional attributes of Los Angeles.

Centrality Type	Attribute Code	Attribute Plaintext	Exceptionality
betweenness	reside-5	Currently lives in a rented hotel or rooming house	0.3333
	usedi	Has used a drug not otherwise listed on the survey	0.3333
	sexid2-5	Does not have sex only with women	0.3333
	racee	Identifies as a race not otherwise specified by the survey	0.2667
closeness	sexid2-5	Does not have sex only with women	0.4000
	usedi	Has used a drug not otherwise listed on the survey	0.3333
	racee	Identifies as a race not otherwise specified by the survey	0.2667
	reside-5	Currently lives in a rented hotel or rooming house	0.2667
eigenvector	racee	Identifies as a race not otherwise specified by the survey	0.4667
	usedi	Has used a drug not otherwise listed on the survey	0.2667
	reside-5	Currently lives in a rented hotel or rooming house	0.2667

Similar to Chicago, the closeness centrality in Fig 3B provides a comprehensive set of nodes surrounding the center-most node in the component, while in Fig 3A betweenness centrality focuses on the nodes along a path between two branches. The attributes labeled in Fig 3 indicate attributes that are significant to that particular node. These attributes may not necessarily be shared between other central nodes, and therefore may not listed within Table 2. Looking at the prevalence of the attribute ‘reside-5’, in which the respondent currently rents a room in a hotel or rooming house, one might conclude that central nodes are less likely to have a consistent living situation, a marker for potential homelessness. The attribute ‘used-i’ indicates that the respondent has used a drug not otherwise listed on the survey. This attribute was also present in each centrality type, indicating higher importance to the overall structure of the network. The attribute ‘sexid2-5’, which was identified as exceptional by betweeness and closeness centralities indicates that the respondent does not have sexual contact exclusively with women.

Fig 4 shows a single component within the Raleigh-Durham referral network. Like both Chicago and Los Angeles, Fig 4B shows that closeness centrality was effective at identifying the nodes around the center-most point in the component. The nodes identified by betweenness centrality are shown in Fig 4A and eigenvector centrality in Fig 4C.

Key attributes on the component highlighted in Fig 4 include marital status, testing positive for gonorrhea, and having graduated from a 4 year university. When looking at Table 3, we notice a much larger number of attributes marked by high exceptionality. Some attributes, such as ‘tmode-5’ and ‘reside-6’ appear across multiple types of centrality, indicating a shared number of central nodes and attributes.

**Table 3:** Exceptional attributes of Raleigh-Durham.

Centrality Type	Attribute Code	Attribute Plaintext	Exceptionality
betweenness	reside-6	Currently lives in a shelter, boarding, or halfway house	0.3333
	slept-2	Slept in a neighborhood, but not a home last week	0.3333
	usedc	Has used heroin and cocaine together	0.2667
closeness	usedc	Has used heroin and cocaine together	0.4000
	usedh	Has used sedatives without a prescription	0.4000
	tmode-5	Walks as a primary form of transportation	0.4000
	usedb	Has used methamphetamines	0.3333
	reside-6	Currently lives in a shelter, boarding, or halfway house	0.3333
	drink1	Has drank alcohol more than average in the past 30 days	0.2667
	slept-3	Has mostly slept in a neighborhood within 20 miles from home	0.2667
	usedf	Has used heroin	0.2667
	slept-2	Slept in a neighborhood, but not a home last week	0.2667
	mstat-5	Currently Divorced	0.2667
eigenvector	tmode-5	Walks as a primary form of transportation	0.4000
	reside-6	Currently lives in a shelter, boarding, or halfway house	0.3333
	rdsq9-1	Has known their recruiter for a very short time (days)	0.3333
	sexid1-9	Does not identify themselves sexually	0.2667
	slept-3	Has mostly slept in a neighborhood within 20 miles from home	0.2667
	slept-2	Slept in a neighborhood, but not a home last week	0.2667

As seen in Table 3, Raleigh-Durham had many more exceptional attributes than other cities. In particular, closeness centrality identified a total of 10 exceptional attributes. The topic of these attributes varies, but several themes seem to appear. Features ‘usedc’, ‘usedh’, ‘usedb’, ‘drink1’, and ‘usedf’ relate to substance abuse of heroin, cocaine, sedatives, alcohol, and others. Attributes ‘reside-6’, ‘tmode-5’, ‘slept-2’, and ‘slept-3’ also appear across multiple centrality types and indicate that the respondent has an inconsistent living situation, ranging from living in a shelter, boarding house, or sleeping in a neighborhood near their home.

## Discussion

In most cases, even seemingly minor attributes identified by exceptional centrality values can be linked back to issues associated either with living with HIV or with the transmission of HIV. Several of the most important examples are discussed below. These examples show that our centrality selection method is able to confirm existing research in a number of cases, as well as to identify new attributes for intervention.

In both Chicago and Raleigh-Durham, martial separation and divorce were found at high rates among nodes with high betweenness and closeness centrality. The relationship between divorce and HIV has been studied in some contexts, such as [47], in which it is is speculated that divorce can be used as a protective mechanism against a spouse who engages in risky sexual behavior, and also that divorce rates should increase in response to risky behaviors in populations with increasing rates of HIV. Both of these situations are true of the Chicago population. Other studies have found high rates of divorce among the HIV population generally [48, 49] and HIV-discordant couples specifically [50]. This is an area where interventions by social workers providing help for couples living with HIV would provide great benefit.

Eigenvector centrality identified several members of a Hispanic community in Chicago based on the their connections to each other. Hispanic communities have been extensively studied in relation to HIV. In particular, it has been speculated that strong social networks have been underused as a resource for spreading prevention information [51], for increasing individual participation in testing [52], and for decreasing risky sexual behaviors [53]. A classic RDS study of Latino men by Ramierez [54] demonstrates that self-protection by Latino men is increased with participation in community involvement activities.

An individual high centrality node in Raleigh reported testing positive for gonorrhea. New HIV diagnoses have been associated with gonorrhea diagnoses [55], particularly among MSM [56]. On the other hand, PrEP has resulted in an outbreak of STDs independent of HIV, including chlamydia [57] and syphillis [58] in addition to gonorrhea.

In Raleigh, the attribute of ‘Age of first sex lower than average’ was detected by eigenvector centrality. In particular, if early sex was the result of sexual abuse, associations have been made with HIV. Child sexual abuse has been shown to lead to risky sexual behavior in both heterosexual men [59] and MSM [60], and is associated with a higher probabilty of being HIV-positive [61].

In Chicago and Raleigh-Durham, participants who walk as a primary form of transportation show exceptional centrality scores. There is research showing that transportation vulnerability is a major issue for HIV-susceptible populations. In particular, transportation vulnerable HIV-positive individuals do not have easy access to care services [62] and are less likely to maintain viral suppression [63]. It has been suggested that providing transportation assistance to susceptible populations decreases the risk of contracting HIV [64]. Our research lends support to this idea, and suggests that it should be pursued further.

Across all three cities, we find that two major themes appear. Firstly, central nodes appear to have higher rates of inconsistent living situations. Attributes such as ‘reside-5’ which describes respondents living in rented hotels or rooming homes, and ‘slept-3’ which describes respondents sleeping in a neighborhood within 20 miles of home are both indicators of higher risks of homelessness in these populations. Studies have found that HIV-positive homeless people had more sex partners, were more likely to exchange sex for money or drugs, and had more unprotected sex than those with homes [65]. Unstable housing has been associated with an increased risk of acquiring HIV for youth [66], and in [67] it is stated that the HIV-positive homeless are 3.84 times more likely to have incomplete viral suppression than the housed. In a San Francisco study, homelessness at diagnosis of HIV was associated with a higher probability of death [68]. The strength of the centrality results for this attribute, combined with the seriousness of the related research, suggest that any interventions by government or charities to reduce homelessness will have an impact on the spread of HIV.

Secondly, nodes identified by the centrality algorithms were more prone to substance abuse. Attributes such as ‘usedi’ which indicated the respondent used a drug not otherwise listed, and ‘usedc’ which indicated the user had used heroin and cocaine together are examples of the several attributes identified by the centrality algorithms that describe the respondent’s substance abuse. Drug use has been suggested as a factor in HIV transmission, although there is debate as to whether sexual or parenteral risk is greater for PWIDs [69]. Other evidence suggests drug use as a causal risk factor for HIV [70]. Due to the connection between drug use and HIV transmission, drug use treatment has been proposed as an HIV prevention strategy [71]. This research supports that proposition.

## Conclusion

This paper discussed the use of centrality based network algorithms to identify biomarkers for the spread of HIV in high risk communities. We utilized the SATHCAP dataset to create a network of referrals across three US cities based upon sexual activity and drug use—behaviors known to transmit HIV. To determine the central, important nodes in these networks, we calculated three forms of network centrality: betweenness, closeness, and eigenvector. By identifying the key attributes of the discovered central nodes, we provide a clear aim for targeted intervention campaigns to both prevent HIV infection and improve the lives of those infected.

The methodology was shown to be successful both in finding established biomarkers, and in identifying new areas for further research. Across the central nodes of the SATHCAP referral network, we found minor themes such as divorce and diagnosis of other STDs. Major themes were also found, such as uncertainty in living situation and substance use. Attributes with high occurrence and relatively small amounts of related research, such as transportation vulnerability, were seen as potential areas for further investigation.

There are many RDS surveys devoted to obtaining biological and behavioral information about HIV populations [72]. Conditions in the world and related behaviors change quickly [73, 74]. This methodology for creating networks from RDS survey data is not exclusive to the SATHCAP dataset. By creating networks where respondents are represented as nodes and recruitments as edges, this methodology can be duplicated on similar datasets. Nodes identified as central to the structure of the RDS network are more likely to have a pronounced impact on the spread of HIV, and make good targets for focused intervention campaigns. Our research found several relevant attributes, and analyses of additional surveys are likely find more.
